# The Prevalence of Malnutrition and Sarcopenia and the Relationship with Inflammation and Anemia Among Community-Dwelling Older Adults: A Preliminary Cross-Sectional Study

**DOI:** 10.3390/geriatrics9060146

**Published:** 2024-11-07

**Authors:** Kornanong Yuenyongchaiwat, Chareeporn Akekawatchai, Khaimuk Changsri

**Affiliations:** 1 Physiotherapy Department, Faculty of Allied Health Sciences, Thammasat University, Pathumthani 12120, Thailand; 2 Thammasat University Research Unit for Physical Therapy in Respiratory and Cardiovascular Systems, Thammasat University, Pathumthani 12120, Thailand; 3 Medical Technology Department, Faculty of Allied Health Sciences, Thammasat University, Pathumtani 12120, Thailand; 4 Thammasat University Research Unit in Diagnostic Molecular Biology of Chronic Diseases Related to Cancer (DMB-CDC), Pathumthani 12120, Thailand

**Keywords:** sarcopenia, malnutrition, anemia, prevalence, older people

## Abstract

**Background**: Older people are more likely to have poor nutrition and low muscle mass, which leads to poor physical performance and anemia, resulting in a poor quality of life and risks to mobility and mortality. Furthermore, malnutrition may, in part, raise the level of inflammatory biomarkers as well as muscle catabolism. Moreover, a range of indices related to systemic inflammation, obtained from routine complete blood count (CBC) tests, have been applied to inflammation markers. However, these biomarkers remain insufficiently addressed in the evidence supporting the presence of sarcopenia and malnutrition. This study aimed to explore sarcopenia in terms of malnutrition, anemia, and inflammation among Thai community-dwelling older people. **Methods**: This study enrolled community-dwelling older people aged 60 years and above. All participants were requested to complete a questionnaire assessing for sarcopenia (SARC-F) and nutritional status using the mini nutritional assessment (MNA). In addition, blood samples were obtained for the CBC test. Logistic regression analysis explored the risk of sarcopenia, CBC, and malnutrition status. **Results**: Of 126 older people (aged 62–88 years) enrolled, 12 individuals (9.52%) had sarcopenia. Furthermore, 34.9% and 5.56% of the participants were demonstrated to have anemia and malnutrition, respectively. Nutrition status was positively associated with hemoglobin levels (*r* = 0.241, *p* = 0.007) and negatively related to SARC-F scores (*r* = −0.190, *p* = 0.034). Older people with anemia show an increased risk of malnutrition at an odds ratio (OR) of 3.375. Moreover, individuals with anemia were at a higher risk of developing sarcopenia (OR 4.982) than those with no anemia. However, individuals with a high level of inflammatory markers, e.g., a high systemic inflammatory response index (SIRI) and monocyte-to-lymphocyte ratio (MLR), had a higher risk of sarcopenia than those with low SIRI and MLR values. The systemic immune–inflammation index (SII) and platelet-to-lymphocyte ratio (PLR) were also positively associated with SARC-F scores. **Conclusions**: The association between sarcopenia, malnutrition status, and anemia might overlap in clinical manifestation. In addition, future research directions regarding the utility of routine CBC testing should focus on sarcopenia and malnutrition status.

## 1. Introduction

Poor nutrition and sarcopenia are common health problems in older people, and these might result in decreased physical performance and quality of life [1,2]. Reportedly, the prevalence of sarcopenia and poor nutrition has been found to vary depending on the assessment for sarcopenia or malnutrition and on the study settings (i.e., in the community, hospital). The prevalence of sarcopenia and malnutrition according to the European Working Group on Sarcopenia in Older People (EWGSOP2) was 19–46% and 51–64%, respectively, among geriatric inpatients [1,2]. According to the criteria established by the Asian Working Group for Sarcopenia 2019, the prevalence of sarcopenia and malnutrition in older individuals who attended a single daycare was 87.1% and 40.3%, respectively [3]. Older Thai adults living in communities, however, only had a malnutrition prevalence of 5.6% to 9.8% [4]. It is clear that older adults in hospitals or long-term care facilities have a higher prevalence of malnutrition and a higher risk of developing sarcopenia than those who live in the community.

Anemia, defined as the presence of low hemoglobin levels, is common in older people. Anemia is a biomarker for adverse health outcomes such as malnourishment and the risk of sarcopenia [5,6,7,8]. Anemia in older people has a wide range of etiologies, including a lack of nutrients (such as ion, vitamin B_12_, and folate) and chronic inflammatory diseases. While nutritional deficiency causes a lack of materials essential for erythropoiesis, hyperinflammatory states typically found in both anemia of inflammation (AI) and anemia of chronic disorders (ACD) in the elderly possess several different pathways leading to anemia. The inflammatory state causes increased production of hepcidin in the liver, increased iron retention in the reticuloendothelial system (RES), and insufficient production of erythropoietin (EPO), leading to inhibition of erythropoiesis. Inflammation also promotes the destruction of red blood cells by increasing the phagocytosis of aging erythrocytes [9,10]. Low hemoglobin levels or anemia have been shown to be related to an increase in inflammation or hyperinflammation in older people [11]. Several studies reported that higher levels of inflammatory biomarkers were noted in older people with sarcopenia compared to those without sarcopenia [12,13]. Thus, loss of muscle mass or sarcopenia and malnutrition, commonly associated with adverse health outcomes, might be related to inflammation [14]. It is also found that malnutrition in older people may, in part, account for an increase in inflammatory cytokine stimulation and activate muscle catabolism; therefore, fatigue or physical weakness can be attributed to these factors [15,16]. Recently, a variety of indexes associated with systemic inflammation from routine complete blood count (CBC) tests have been used to indicate inflammatory biomarkers. However, these biomarkers have not been adequately studied in older adults. In other words, the routine CBC has not been fully elucidated [17,18]. Further, inadequate oxygen transportation leads to decreased oxygen delivery to the tissues and might result in physical weakness, reduced muscle strength, and poor physical performance [19,20]. Hence, the study aimed to explore sarcopenia and malnutrition in community-dwelling older people and determine the relationships with hematologic parameters of anemia and inflammation.

## 2. Methods

### 2.1. Study Design

A preliminary study was conducted with a cross-sectional study of 126 older participants. Ambulatory older people aged 60 years and above who were living independently in the community were enrolled in the study. Participants were excluded from the study if they had a resting heart rate exceeding 120 beats per minute, uncontrolled resting blood pressure (defined as greater than 180/120 mmHg), uncontrolled blood glucose levels, or a history of cardiovascular disease (such as stroke or coronary artery disease) or psychiatric disorders. Additionally, individuals with musculoskeletal problems such as knee osteoarthritis were also excluded from participation. All participants were required to provide blood samples for routine CBC testing, which measured blood components including red blood cells, white blood cells, and platelets using an automated blood cell counter (HORIBA medical Yumizen H1500 analyzer, Kyoto, Japan).

### 2.2. Study Protocol

All participants were required to be assessed for sarcopenia and malnutritional status. Screening for sarcopenia was conducted using a cut-off point of 4 on the SARC-F questionnaire; participants with screening questionnaire for sarcopenia scores of ≥4 were considered to be at risk for sarcopenia [21,22,23]. The scores ranged from 0 to 10, with higher scores indicating a higher risk of sarcopenia. Malnutrition status was assessed by the mini nutritional assessment (MNA), which is a validated instrument commonly used for assessing nutritional status in older people [24,25,26]. The MNA consists of six items presented as a checklist on malnutrition status, weight loss, mobility, stress, neuropsychological problems such as depression and dementia, and body mass index within three months, rated from 0 to 14. A score of 12 or more was interpreted as normal nutrition, a score of 7 or less was interpreted as malnutrition, and a score between 8 and 11 was considered to indicate a risk of malnutrition [25,27,28].

Data collected from CBC results included hemoglobin levels, the number of red blood cells (RBC), platelets, and white blood cells (WBC), with differential counts of neutrophils, monocytes, and lymphocytes. CBC-derived parameters, the systemic inflammatory response index (SIRI), the systemic immune–inflammation index (SII), the neutrophil–lymphocyte ratio (NLR), the platelet–lymphocyte ratio (PLR), and the monocyte–lymphocyte ratio (MLR) were calculated as follows: SIRI was defined as neutrophil count x monocyte count/lymphocyte count; the SII was defined as platelet count x neutrophil count/lymphocyte count, the NLR was defined as neutrophil count/lymphocyte count, PLR was defined as platelet count/lymphocyte count, and MLR was defined as monocyte count/lymphocyte count [29,30]. Hemoglobin levels less than 12 g/L for females and less than 13.0 g/L for males were categorized as anemia [31].

### 2.3. Data Analysis

Statistical analysis was conducted using SPSS program version 24.0. Descriptive data were displayed as mean ± SD or percentages. ANOVA and independent *t*-tests were used for the comparison between sarcopenia and no-sarcopenia groups, based on SARC-F, or malnutrition and no-malnutrition groups, where appropriate. Further, Pearson correlation and logistic regression analysis were used to examine the relationships with sarcopenia, malnutrition, and anemia. Statistical significance was set at a *p*-value less than 0.05.

## 3. Results

### 3.1. Characteristics of Studies

A total of 126 community-dwelling older people aged 62–88 years were enrolled in the study. Of these, 12 (9.52%) had sarcopenia, with a SARC-F score of ≥4. Additionally, there were seven (5.56%) participants with MNA scores of <7, which were defined as malnutrition. In addition, 34.90% of the older adults were classified as having anemia according to criteria established by the World Health Organization (WHO) [31]. Table 1 displays the descriptive variables for sarcopenia and no sarcopenia assessed using the SARC-F. Statistically significant differences between the presence of sarcopenia and no sarcopenia based on SARC-F were noted in terms of inflammatory status (i.e., SIRI and MLR); high SIRI and MLR values indicated a high risk for sarcopenia (*p* < 0.05).

According to malnutrition status, the study revealed that older people who were well-nourished had higher haemoglobin levels than those at risk of malnutrition and with malnutrition (see Table 2). In addition, participants without sarcopenia were well-nourished compared to those at risk of undernutrition (*p* < 0.002).

### 3.2. The Relationships with Hematologic Parameters of Anemia and Inflammation

The results revealed that nutrition status was positively associated with hemoglobin levels (*r* = 0.241, *p* = 0.007). This suggests that participants with malnutrition status had low oxygen transportation and a high risk of sarcopenia. Interestingly, the SARC-F scores were inversely correlated to hemoglobin levels (*r* = −0.190, *p* = 0.034), but passively associated with platelet levels (*r* = 0.232, *p* = 0.009), SII (*r* = 0.182, *p* = 0.041), and PLR (*r* = 0.212, *p* = 0.017). Further, partial correlation reported that these relationships survived adjustments for age and sex (see Table 3).

Finally, to determine the risk of developing sarcopenia or malnutrition, univariate analysis was conducted (Table 4). Anemia emerged as a significant risk factor for sarcopenia and malnutrition (defined as MNA < 11 scores). Older people with anemia showed an increased risk of malnutrition at an odds ratio (OR) of 3.375 (95% confidence interval (CI) 1.570–7.255, *p* = 0.002). Further, individuals with anemia were at higher risk of developing sarcopenia (OR 4.982, 95% CI 1.219–20.360, *p* = 0.025).

## 4. Discussion

The purpose of this study was to explore the prevalence of sarcopenia and malnutrition status in older people dwelling in the community and to evaluate their relationship with hematologic markers of inflammation and anemia. Of 126 older people examined, 9.52% and 44.40% were at risk of developing sarcopenia (defined as SARC-F ≥ 4) and malnutrition (defined as MNA < 11 scores), respectively. Further, the prevalence of anemia was demonstrated in 44 participants aged older than 60 years; these participants displated hemoglobin levels less than 13 g/dL in males and 12 g/dL in females [31].

Changes in multiple physiological settings associated with sarcopenia and malnutrition have been described [9,32,33]. This study demonstrated a significant correlation between malnutrition (defined using MNA) and risk of sarcopenia (measured using SARC-F). This finding supports the notion that protein–energy malnutrition is often observed and leads, together with other covariates, to sarcopenia in older adults [33]. Lack of nutrients essential for erythropoiesis, such as iron, folate, and/or vitamin B_12_, are also major causes of anemia in older adults [9]. Our data also suggested that anemia, assessed by low levels of hemoglobin, was a risk factor for both malnutrition and sarcopenia. The analysis indicated that hemoglobin levels were associated with MNA, and correlated with SARC-F. Low hemoglobin levels indicate insufficient nutrient intake, which leads to impaired protein synthesis and plays a role in reductions in muscle mass and muscle strength; therefore, this might result in the development of sarcopenia [34]. Thus, anemia could be a major physiological alteration associated with the risk of sarcopenia and malnutrition in this study group.

Interestingly, apart from nutritional anemia, anemia of inflammation (AI) or anemia of chronic disease (ACD) are also considered to be prevalent in older adults [10]. Our analysis indicated a significant correlation between inflammation index, SII, platelets, and PLR with SARC-F. These findings are consistent with a previous study showing that platelet count and platelet-to-WBC ratios (PWR) were associated with sarcopenia [35]. Although mechanisms underlying the effect of platelets on sarcopenia remain unclear, platelets are demonstrated to be an initiator of vascular inflammation and remodeling, which might disturb oxygen and nutrient supply in muscle cells by interfering with microcirculation and the vascular endothelium [36]. Several studies have supported the association of inflammation and anemia with sarcopenia [33,35]. The combination of inflammation and physiological impairments, such as decreased hemoglobin levels, might be associated with muscle mass and physical function [31]. Steinmeyer et al. [37] found that hemoglobin levels are related to frailty in older people aged 65 years and above. Additionally, frailty risks are associated with inflammation (i.e., C-reactive protein: CRP) and malnutritional status (i.e., MNA). Further, low hemoglobin levels are correlated to malnutrition status. They proposed that the pathway to frailty status is displayed in oxygen transportation, inflammation, and malnutrition status. The overlap between sarcopenia and frailty status is presented in several studies [38,39,40]. Therefore, sarcopenia, malnourishment, anemia, and inflammation are interrelated, and there are partial overlaps between these factors.

This study has several limitations. Sarcopenia was assessed using SARC-F, which is a screening tool used to investigate or assess the risk for sarcopenia in the community [41]; therefore, a diagnosis of sarcopenia should be informed by measuring muscle mass, physical performance, and muscle strength [41]. Confounding factors, such as comorbidities and health status, were not recorded. Further, these participants were ambulatory; hence, the results might not be generalizable to older people. Given the small sample size of the present study, owing to the COVID-19 pandemic and lockdown, future studies with larger samples should be conducted to ascertain the relationship between these factors.

In conclusion, ooverlaps in the clinical presentation of sarcopenia, malnutrition, inflammation, and anemia may, in part, explain the link in the relationships with sarcopenia in older Thai people. However, the assessment of routine CBC (e.g., NLR, PLR, and MLR) needs to be explored in further study. Therefore, therapists or health care providers should consider the combination of muscle mass, malnutrition, and laboratory investigations in older people, and take appropriate interventions to modify the risk of adverse health outcomes.

## Figures and Tables

**Table 1:** Characteristics of older Thai adults based upon SARC-F (n = 126).

	Total (N = 126) Mean ± SD	SARC-F (≥4 Scores) (N = 12) Mean ± SD	SARC-F (<4 Scores) (N = 114) Mean ± SD	*p*-Value
Age (yrs)	68.71 ± 5.49	69.33 ± 5.25	68.65 ± 5.53	0.683
BMI	25.67 ± 4.26	25.77 ± 4.05	25.66 ± 4.30	0.936
Sex ^a^				0.686
Female (%)	102	9 (8.82%)	93 (91.18%)	
Male (%)	24	1 (4.17%)	23 (95.83%)	
RBC	4.61 ± 0.59	4.42 ± 0.88	4.63 ± 0.55	0.241
Hb	12.41 ± 1.27	11.79 ± 1.61	12.48 ± 1.21	0.074
WBC	7.70 ± 2.09	7.97 ± 1.62	7.67 ± 2.14	0.639
Plt	270.38 ± 62.26	295.92 ± 64.34	267.69 ± 61.71	0.136
Neu	51.39 ± 11.73	55.59 ± 14.13	50.95 ± 11.44	0.193
Lymp	38.51 ± 10.49	33.78 ± 12.03	39.00 ± 10.24	0.101
Mono	4.89 ± 2.42	6.03 ± 2.89	4.77 ± 2.35	0.087
SIRI	7.20 ± 4.68	10.15 ± 5.42	6.89 ± 4.51	0.021
SII	417.66 ± 266.47	538.39 ± 221.87	404.95 ± 268.41	0.099
NLR	1.53 ± 0.82	1.96 ± 1.02	1.49 ± 0.79	0.060
PLR	7.65 ± 3.19	9.33 ± 2.48	7.48 ± 3.21	0.055
MLR	0.14 ± 0.08	0.18 ± 0.08	0.13 ± 0.08	0.029
MNA	11.25 ± 2.15	10.33 ± 1.67	11.38 ± 2.06	0.093

^a^ Analyzed using the chi-square test. BMI: Body mass index, RBC: red blood cell, Hb: haemoglobin, WBC: white blood cell, Plt: platelet, Neu: neutrophil, Lymp: lymphocyte, Mono: monocyte, SIRI: systemic inflammatory response index, SII: systemic immune–inflammation index, NLR: neutrophil-to-lymphocyte ratio, PLR: platelet-to-lymphocyte ratio, MLR: monocyte-to-lymphocyte ratio, MNA: mini nutritional assessment.

**Table 2:** Characteristics of older Thai adults based upon the mini nutritional assessment (n = 126).

	Total (N = 126)	Malnourished (N = 7)	Risk of Malnutrition (N = 49)	Well-Nourished (N = 70)
Age (yrs)	68.71 (5.49)	71.57 (5.80)	68.53 (5.68)	68.56 (5.33)
Sex ^a^				
Female (N = 102)	102	4 (3.92%)	40 (39.22%)	58 (56.86%)
Male (N = 24)	24	3 (12.50%)	9 (37.50%)	12 (50.00%)
RBC	4.61 (0.59)	4.29 (0.68)	4.58 (0.71)	4.67 (0.47)
Hb	12.41 (1.27)	11.51 (1.33) ^b^*	12.10 (1.45) ^a^*	12.72 (0.59) ^a^*^,b^*
WBC	7.70 (2.09)	8.61 (2.63)	7.37 (1.76)	7.84 (2.23)
Plt	270.38 (62.26)	278.57 (61.10)	277.55 (67.68)	264.54 (58.58)
Neu	51.39 (11.73)	43.31 (13.46)	52.42 (12.82)	51.47 (10.58)
Lymp	38.51 (10.49)	46.47 (11.30)	37.03 (11.90)	38.74 (9.03)
Mono	4.89 (2.42)	3.84 (2.56)	4.97 (2.25)	4.94 (2.53)
SIRI	7.20 (4.68)	4.96 (7.52)	7.61 (4.29)	7.13 (4.62)
SII	417.66 (266.47)	302.21 (209.97)	468.84 (315.08)	393.37 (227.95)
NLR	1.53 (0.82)	1.07 (0.70)	1.69 (1.02)	1.47 (0.65)
PLR	7.65 (3.12)	6.42 (2.44)	8.34 (3.67)	7.30 (2.81)
MLR	0.14 (0.08)	0.10 (0.11)	0.14 (0.07)	0.14 (0.08)
SARC-F	1.10 (1.70)	1.43 (1.40)	1.71 (2.02) ^a^**	0.64 (1.08) ^a^**

^a,b^ Significant mean differences. * *p* < 0.05, ** *p* < 0.01. RBC: red blood cell, Hb: hemoglobin, WBC: white blood cell, Plt: platelet, Neu: neutrophil, Lymp: lymphocyte, Mono: monocyte, SIRI: systemic inflammatory response index, SII: systemic immune–inflammation index, NLR: neutrophil-to-lymphocyte ratio, PLR: platelet-to-lymphocyte ratio, MLR: monocyte-to-lymphocyte ratio.

**Table 3:** Nutrition and risk of sarcopenia and the correlation with haematologic profiles in older people.

	SARC-F *r* (*p*-Value)	RBC *r* (*p*-Value)	Hb *r* (*p*-Value)	WBC *r* (*p*-Value)	Plt *r* (*p*-Value)	Neu *r* (*p*-Value)	Lymp *r* (*p*-Value)	Mono *r* (*p*-Value)	SIRI *r* (*p*-Value)	SII *r* (*p*-Value)	NLR *r* (*p*-Value)	PLR *r* (*p*-Value)	MLR *r* (*p*-Value)
MNA	−0.255 (0.004)	0.085 (0.343)	0.241 (0.007)	0.039 (0.664)	−0.076 (0.395)	0.102 (0.254)	−0.085 (0.345)	0.069 (0.444)	0.042 (0.642)	0.004 (0.961)	0.016 (0.860)	−0.026 (0.775)	0.042 (0.643)
SARC-F		−0.071 (0.432)	−0.190 (0.034)	0.167 (0.061)	0.232 (0.009)	0.125 (0.162)	−0.130 (0.148)	0.078 (0.384)	0.151 (0.092)	0.182 (0.041)	0.159 (0.079)	0.212 (0.017)	0.122 (0.173)
MNA #	−0.259 (0.004)	0.105 (0.246)	0.283 (0.001)	0.030 (0.738)	−0.102 (0.258)	0.085 (0.345)	−0.072 (0.426)	0.075 (0.407)	0.028 (0.758)	−0.016 (0.861)	0.000 (0.861)	−0.049 (0.591)	0.039 (0.665)
SARC-F #		−0.062 (0.496)	−0.187 (0.037)	0.169 (0.061)	0.236 (0.008)	0.117 (0.195)	−0.125 (0.165)	0.086 (0.341)	0.150 (0.097)	0.183 (0.042)	0.155 (0.086)	0.214 (0.017)	0.125 (0.168)

# Partial correlation by controlling for age and sex. RBC: red blood cell, Hb: hemoglobin, WBC: white blood cell, Plt: platelet, Neu: neutrophil, Lymp: lymphocyte, Mono: monocyte, SIRI: systemic inflammatory response index, SII: systemic immune–inflammation index, NLR: neutrophil-to-lymphocyte ratio, PLR: platelet-to-lymphocyte ratio, MLR: monocyte-to-lymphocyte ratio, MNA: mini nutritional assessment.

**Table 4:** Univariate analysis of risk associations for sarcopenia and malnutrition in older people.

	Odds Ratio	95% CI for OR	*p*-Value
Malnourished or at risk of malnutrition			
	Reference group NLR (≤2.8)		
NLR (>2.8)	4.080	0.790 to 21.063	0.093
	Reference group MLR ≤ 0.29		
MLR (>0.29)	1.259	0.172 to 9.233	0.821
	Reference group SIRI (≤1.25)		
SIRI (>1.25)	1.033	0.359 to 2.972	0.952
	Reference group normal		
Anemia	3.375	1.570 to 7.255	0.002
Risk of sarcopenia according to SARC-F			
	Reference group NLR (≤2.8)		
NLR (>2.8)	4.583	0.793 to 26.478	0.089
	Reference group MLR ≤ 0.29		
MLR (>0.29)	4.185	0.394 to 44.449	0.235
	Reference group SIRI (≤1.25)		
SIRI (>1.25)	1.033	SIRI (>1.25)	1.033
	Reference group normal		
Anemia	4.982	1.219 to 20.360	0.025

SIRI: Systemic inflammatory response index, NLR: neutrophil-to-lymphocyte ratio MLR: monocyte-to-lymphocyte ratio.

## Data Availability

The datasets analyzed during the current study are available from the corresponding author upon reasonable request.
